# Older adults’ perspectives on physical activity and sedentary behaviour within their home using socio-ecological model

**DOI:** 10.1371/journal.pone.0294715

**Published:** 2023-11-20

**Authors:** Naureen Akber Ali Meghani, Joanne Hudson, Gareth Stratton, Jane Mullins

**Affiliations:** 1 Applied Sports Technology, Exercise and Medicine (A-STEM) Research Centre, Swansea University, Bay Campus, Swansea, United Kingdom; 2 Professor of Exercise and Sport Psychology, Applied Sports Technology, Exercise and Medicine (A-STEM) Research Centre, Swansea University, Bay Campus, Swansea, United Kingdom; 3 Chair in Paediatric Exercise Science, Applied Sports Technology, Exercise and Medicine (A-STEM) Research Centre, Swansea University, Bay Campus, Swansea, United Kingdom; 4 College of Human and Health Sciences, Swansea University, Singleton Campus, Swansea, United Kingdom; PLOS: Public Library of Science, UNITED KINGDOM

## Abstract

**Background:**

There are few studies that focus explicitly on the impact of the home environment on older adults’ sedentary behaviour (SB) and physical activity (PA) using the socio-ecological model (SEM). This study aims to investigate older adults’ PA and SB within the home environment integrating the SEM as a theoretical framework.

**Methods:**

A qualitative exploratory research design was employed to conduct 33 in-depth interviews (IDIs) and five focus group (FGs; n = 16) with multi-diverse ethnic older adults (mean age 72±5 years). Using reflexive thematic analysis themes were generated from the data set and were interpreted using the SEM.

**Results:**

The findings indicate that different levels of the SEM had an impact on older adults’ PA and SB. These include the 1) Individual level: Attitude, perception and motivation 2) Interpersonal level: Family and Friends: a motive to remain active 3) Organisational level: healthcare institutes, 4) Community level factors: Significance of social groups, 5) Physical Environment: Microenvironment and 6) Policy level factors (lockdown restrictions and healthcare system). This model can be utilised to foster activity within the home by focusing on the facilitators and barriers identified at each of these levels of influence.

**Conclusion:**

The study findings suggest that modifying PA and SB in the home environment is complex and is influenced across different levels of the SEM. Therefore, a holistic approach is required that integrates these multiple influences. This understanding can inform the design of interventions that seek to optimize PA and minimize SB within the home environment.

## Introduction

The health and wellbeing of the ageing population has become one of the most significant global challenges [1]. The 2019 Revision of World Population Prospects estimates that by 2050, one in six individuals will be aged above 65 years: an increase from one in eleven in 2019. Also, the number of individuals aged 80 years or over will triple [2]. However, this increase in life expectancy is not correlated with a rise in quality of life and health, as functional losses, psychological impairment and loneliness increase with age [3–5]. Subsequently, medical costs to treat age-related conditions and illnesses generate the highest financial burden for the majority of nations around the world [3]. To lower healthcare expenditure, it is essential to help older adults maintain their independence and an active lifestyle to promote mental and physical health [6–8]. Recommendations from the World Health Organization (WHO) state that older adults should engage in 150–300 minutes per week of moderate intensity activity or 75–150 minutes per week of vigorous intensity activity, or some equal combination of moderate and vigorous intensity aerobic activity, and on two or more days per week, do muscle-strengthening activity (such as strength and resistance training) to promote health and well-being [9]. However, current estimates indicate that only two in five (39%) individuals aged 75 years and above were active in comparison with 7 in 10 (69%) 16 to 24 year olds [10].

Moderate-to-vigorous physical activity (PA) is an essential behaviour for promoting health and well-being at all ages [11–14] where a substantive body of evidence demonstrates beneficial effects for physical and mental health and well-being [13, 15–17]. It is estimated that a greater level of PA reduces the risk of type 2 diabetes by up to 40%, of cardiovascular disease by up to 35%, of depression and falls by up to 30%, of back and joint pain by up to 25% and of breast and colon cancer by up to 20% [18]. Moreover, a number of PA based interventions (exercise and walking), for falls prevention [19], slowing cognitive decline [20], and balance improvement [21] have shown promising results in randomised clinical trials (RCTs) and systematic reviews. Hence there is compelling evidence to support the promotion of PA for health in adults [22].

Sedentary behaviour (SB), especially when associated with extended durations of sitting, is also a risk behaviour [23]. SB is defined as any waking behaviour exemplified by an expenditure of energy less than or equal to 1.5 metabolic equivalents (METs), when in a sitting, lying or reclining posture (for example, watching television; [24, 25]. SB is prevalent throughout the population, but older adults have been found to be the most sedentary group in the population [26, 27]. Their total of 10 to 14 hours on average of sitting per day places them at a significantly higher risk for developing various non-communicable diseases [28–30]. In contrast, people who minimize their level of SB seem more likely to have improved quality of life [31, 32]. For instance, lower sitting time was related to higher global physical health (GPH) and global mental health (GMH) scores among less sedentary older adults [33]. Similarly higher well-being (positive affect) was strongly related to more PA and less sitting time [34].

Research on older adults found that they spend 70.1% of their time being sedentary at home [35], suggesting that the home environment has a significant impact on older adults’ PA and SB. According to research, the physical home environment can act as a both an enabler and a barrier to older adults’ PA and SB [36–38]. Regardless of the significance of the physical environment of the home, it was also noted that the home environment is socially influenced by the individuals who live there; making the home a dynamic ecological setting [39]. Given this potential diversity of factors influencing PA and SB in the home environment, a useful model for advancing our understanding of older adults’ PA and SB is the socio-ecological model [40]. This model highlights the importance of human-environment interactions as key influencing variables on behaviour [40, 41]. Five levels of influence are suggested by this model: individual traits; age, and motivations, interpersonal variables; family and friends, organisational factors; healthcare institutes, community factors; societal norms, and policy-level factors; laws and policies [40].

The home has great significance in relation to PA and SB as it is the one place that people may control, can express their personal identity, experience autonomy, safety and privacy and which influences the activity level that the individual performs during their daily routines [42, 43]. The home environment in our study refers to the microenvironment within which individuals are residing [44, 45]. Most research has focused on the urban neighbourhood at the organisational level for the SEM, highlighting accessibility to locations, amenities, and services such as recreational opportunities, and public transport, as an important factor for PA [45–51]. Whilst these are significant determinants, they do not account for the microenvironment that the majority of older adults live in and spend the majority of their time [52]. According to a global report of 10 nations [42], the majority of older adults reside independently in their communities rather than in more supported environments (e.g., care facilities or communal residences). Indeed, the majority of older adults (≥ 65 years) in the UK live alone within their indoor home environment [42, 53]. This signifies the importance of making the home environment conducive for older adults to enhance their PA engagement and minimize their SB, in accordance with the diverse layers of the SEM [54].

Given that older adults spend the majority of their time being sedentary at home [35], the indoor home setting is therefore an important context to understand. It has, however, received limited research attention but is a key subject for investigation in PA [55–59] and SB research [35, 54]. Those research studies that have explored the relationship between the home environment and PA and SB have mainly focused on space within the home and garden, fittings and fixtures (stairs, kitchen and bathroom space) to understand older adults’ PA patterns in their home environment [55–59]. However, these studies failed to assess barriers and facilitators to PA and SB using all the levels of SEM that include individual, interpersonal, organisational, community and policy-level factors. As older adults become increasingly dependent on their personal environment, to support their PA, the home and factors associated with it become strong determinants of their PA and SB [42]. Therefore, the primary aim of the present study was to understand the influences on older adults’ PA and SB within the home environment, using the SEM as a theoretical framework. In doing so, this qualitative research will determine the barriers to, and facilitators of, older adults’ PA and SB in their home environments.

## Material and methods

### Study design

The study adopted a qualitative approach to explore the impact of the home environment on older adults’ SB and PA level using the SEM. This qualitative exploratory research design used in-depth interviews (IDIs) and focus group discussions (FGDs) with purposely sampled older adults. Interviews were carried out from 1^st^ August 2022 to 31^st^ December 2022.

IDIs and FGDs were conducted by the first author (NA) who had training and previous experience in carrying out qualitative research interviews. The reason behind conducting FGDs following IDIs is that for some older adults, being familiar with their home environment, could restrict their ability to think critically about ways of optimizing their home setting. A group conversation could provide them with an opportunity to think differently about their familiar setting on hearing different views and experiences.

### Participant inclusion and exclusion criteria

The target population for this study were older (aged ≥ 65 years) men and women living in Swansea, United Kingdom, who lived at home and were able to communicate in English or Urdu. Those who experienced any psychological issues or were unable to cooperate with the research team for the full duration of the project were excluded. We purposively sampled older adults from a diverse ethnic group to collect varying perceptions regarding home environment utilization for PA and SB.

### Setting and recruitment

Recruitment was achieved through local religious and social community groups that organised recreation, leisure and sports activities for diversely ethnic older adults in Swansea. Local coordinators and sub-coordinators acted as gatekeepers as they were approached to enrol older adults using sport, play and community networks. Older adults who were currently or previously affiliated with these organizations or groups were contacted. Approval was requested from the co-ordinators for the first author to conduct a visit to inform older adults about the study. Older adults were requested to express their interest to their corresponding group co-ordinator, sub-co-ordinator or the researcher. Those who responded were given the option for either an in-person (face-to-face) or remote/virtual interview and approached via call (WhatsApp or telephone) or face-to-face or email. Older adults who decided to participate were given participant information sheets, and the lead researcher explained the study’s processes and consent was obtained prior to interview or focus group participation.

### Data collection procedure

The study was approved by an institutional Research Ethics Committee (REC: NM_31-03-22), and following this, interviews were conducted by the primary researcher (NA) at a suitable time for the older adult and upon gaining the older adult’s written consent. A semi-structured interview guide was developed (see S1 File Interview guide) based on the SEM [60] with particular emphasis on the home environment. The guide also covered several factors at different layers of the SEM to explore their effect on older adults’ PA and SB. These include individual-level factors (self-motivation, attitude and motivation), interpersonal level factors (family and friends), organisational level factors (healthcare institutes), community level factors (social groups), physical environment (significance of the home and garden space, physical equipment, electronic media equipment and changing infrastructure within the home) and policy level factors (influence of lockdown, and role of healthcare system). Study participants were questioned about personal, family and community roles in PA and SB, about their home environment itself, including what they like about their home setting, how it was chosen, what kind of physical activities they performed within the home environment, use of garden space and stairs and how the home environment had been or could be altered to promote more PA and reduce SB. Older adults were also asked about the presence of PA equipment within their home and its frequency of use. Following this they were questioned about community services, healthcare services, the role of technology and impact of COVID-19 pandemic on their activity levels. In addition, socio-demographic data (age, gender, level of education, occupation level, ethnicity, and marital status) were collected.

Firstly, all IDIs (n = 33) were conducted online and lasted between 30–40 minutes. Next, FGDs (n = 5) were conducted face-to-face with participants who had not taken part in the IDIs. FGDs were carried out in a private room in a community hall where the religious and community groups met and lasted between 50 and 60 minutes. The male and female distribution for IDIs and FGDs is shown in Table 1. Data collection ceased once data saturation was achieved.

**Table 1:** Study participants who participated in in-depth interviews and focus group discussions.

Data Collection Method	Male	Female	Total
In-depth Interview (IDIs n = 33)	15	18	33
Focus group Discussion (FGDs n = 5)	6	10	16
Total	21	28	49

### Data analysis

All IDIs and FGDs recordings were transcribed, and interviews conducted in Urdu (n = 7) were translated into English. Reflexive thematic analysis (TA) was used to identify patterns and themes in the data [61]. The approach outlined by Braun and Clarke (2022) [62] was used, which combines inductive (driven from data) and deductive (actively looking for determinants influencing PA and SB at home) methods. The first step in the process was familiarisation, which required reading and rereading the transcripts and underlining important data, such as information that was repeated across several interviews, linked to earlier research, and presented a unique finding. Then, using NVivo 12 (NVivo12, QSR), these noteworthy data were coded. After examining the codes and assembling similar codes together, primary themes and sub-themes were created in a hierarchical order. The transcript data were used to consider and verify the names of the final theme. The SEM was then used to deductively examine the data and map the themes to the model’s five levels: individuals, interpersonal/relationships, organisations, community, and policy level.

The researchers implemented the steps suggested by Campbell et al [63] to promote the quality of analysis by first participating in a reflexive process, followed by group discussions of the research team to assure trustworthiness and credibility. In addition, prolonged listening to the interview tapes permitted the researchers to thoroughly engage themselves in the topic and gain a deeper insight into the themes being discussed.

## Results

The mean age of participants was 71.7 ± 5.2 years (57.1% females). On average 39.0% of older adults spent more than two hours a day watching TV, while 84.5% spent more than two hours a day on their mobile phone, computer/laptop or the internet for leisure. A high percentage (79.5%) had a university degree.

Due to the absence of data on income and SES, the 2019 Welsh Index of Multiple Deprivation scores calculated from postcodes were used as a measure of socioeconomic status (SES). Eight areas of deprivation are taken into account when calculating the WIMD scores: health, employment, housing, income, community safety, education, access to services, and the environment [64]. Resulting scores range from 1–1909, with 1909 being the least deprived and 1 the most deprived. Based on the WIMD score, participants were divided into SES tertiles: low-SES (1–636), medium-SES (637–1272), and high-SES (1273–1909) groups as used previously [65]. Based on WIMD scores, 16% of the older adults were categorised as low-SES, 35% as medium-SES and 49% as high-SES. The remaining socio-demographic characteristics of the older adults are provided in Table 2.

**Table 2:** Characteristics of study participants (n = 49).

**Age**	72±5
**Gender**	
Male	28 (57%)
Female	21 (43%)
**Education level**	
No formal education	1 (2%)
Primary School	1 (2%)
Secondary/High School	8 (16%)
College/University	24 (49%)
Postgraduate	15 (31%)
**Occupation level**	
Retired	40 (82%)
Self-employed	9 (18%)
**Ethnicity**	
British	16 (33%)
Pakistani	13 (27%)
African	9 (18%)
Indian	4 (8%)
Bangladeshi	4 (8%)
Irani	2 (4%)
Iraqi	1 (2%)
**Marital status**	
Married	36 (74%)
Separated	1 (2%)
Single	6 (12%)
Widowed	5 (10%)
Divorced	1 (2%)
**Home Status**	
Owned	45 (92%)
Rented	4 (8%)

The subthemes that were identified within the deductive themes derived from the SEM 1) Individual level: Attitude, perception and motivation 2) Interpersonal level: Family and Friends: a motive to remain active 3) Organisational level: healthcare institutes, 4) Community level factors: Significance of social groups, 5) Physical Environment: Microenvironment and 6) Policy level factors (lockdown restrictions and healthcare system). are presented in Table 3.

**Table 3:** Themes, subthemes and quotations of study participants.

Themes	Subthemes	Description	Quotations
**1. Individual level: Attitude, perception and motivation**	Positive attitudes towards PA	Older adults shared that they were motivated to be active to remain mobile, fit, and healthy, avoid pain and maintain mental health and wellbeing.	‘I keep myself mentally and physically busy; sitting down and watching television is not me. I am busy all day.’ (FG1-F-1, Pakistani, High SES)
	Self-perceived health	Declining physical health act as a barrier to continuing physical activity. However, some older adults continued to perform their activity despite their health issues and made attempts to keep themselves moving.	‘I walk throughout the day in my home. It is important for me to be active due to sciatica and arthritis; sitting in one place is not good for me.’ (IDI-22F, Bangladeshi, High SES)
	Positive self-perception	Positive self-perceptions of aging were strongly linked with physical activity. Many older adults shared that positive self-perceptions assist them in being active and doing physical activity.	‘I think age is just a number; I am active and younger mentally. I don’t think myself old…I think I am active I can do everything. If you think you are old, you will never take care of yourself.’ (FG1-F-3, Pakistani, Middle SES)
	Lack of motivation	Many older adults shared that they were keen to be physically active, knowing the benefits of activity for their health, however, lack of motivation prevented them initiating physical activity within their home environment.	‘I have time but it’s laziness that is hindering me to do physical activity, I have to motivate myself to get involved in any physical activity like exercise or yoga at home.’ (IDI-20M, African, Middle SES)
**2. Interpersonal level: Family and Friends: a motive to remain active.**		Family support was an important theme that was identified in this study. Living with the family had a great impact on older adults’ status of being active, functioning and performing their roles and responsibilities.	‘My family member encourages me to do walking within the home or in the garden … my son and daughter walk by my side in the garden, so I don’t fall out during walking.’ (FG1-F-2, Pakistani, Low SES)
		Friends’ influences apparently had a major effect on physical activity and sedentary behaviour, due to the support they give. This also provides an opportunity to have positive perception about themselves as reported by a participant.	‘…my friend helps me to keep myself young because they say we are young and then I also consider myself young. I am still young I will do everything. I never give up.’ (FG1-F-4, Pakistani, Low SES)
**3. Organisational Level: Health care institute**		A key organisational-level theme was the role of health institutes for encouraging older adults to be physically active within their home. Older adults suggested the need for introducing exercise prescription model in the healthcare system as it played a substantial role in maintaining their health- and well-being.	‘I think more time should be invested in natural remedies or physical activities or yoga rather than just giving medications to elderly and making them dependent on them.’ (IDI-13F, Bangladeshi, Middle SES)
		Older adults discussed that the role of healthcare professional advice is very important in directing them to improve their activity level.	‘Other than walking I also do some gentle stretches that are suggested by neurologists. My leg and pelvic muscles are weak so to make them strong I do some kinds of exercise as suggested by the doctor.’ (IDI-13F, Bangladeshi, Middle SES)
**4. Community level: Significance of social groups**		Being part of a social group often provided opportunities for the older adults to be physically active. However, some suggested the need for culture sensitive groups. Some older adults stated that lack of access to Asian community facilities lessened their opportunities to be physically active within community	‘Culture-appropriate activities for elderly people from the west community are required.’ (IDI-21F, Pakistani, High SES) ‘Again, problem is that in Swansea we don’t have an Asian community centre but other cities like Birmingham, Manchester, and Bristol have these facilities.’ (FG5-F-1, British, High SES)
**5. Physical Environment: Microenvironment**	Significance of the home and garden space	Older adults shared that proper space in their rooms helps them to be active and keep moving as they are able to perform different kinds of physical activities within their home environment.	‘My home is spacious, so I like to roam within home rather than sitting on a place.’ (FG2-F-2, Bangladeshi, Low SES)
		Older adults admired their garden and commented that it was a major source of physical activity and rejuvenation.	‘I have a garden where I do some exercises and enjoy gardening. It keeps me fit and moving. I am glad I got a garden that keeps me busy.’ (IDI-12F, British, Middle SES)
	Changing infrastructure within the home space	On inquiring about how to make their home environment supportive of activity, many older adults shared diverse views. Some expressed possible ways to utilize their home gym effectively, while some expressed a desire to buy physical activity equipment. Interestingly, some older adults came up with the idea of designing an indoor swimming pool, dance floor, sport room, and home gym, to optimize their activity, promoting their health and well-being.	‘…a small swimming pool within the home to be active…’ (IDI-11M, Iraqi, Middle SES)
	Physical activity equipment	In terms of availability of physical activity equipment within the home mixed opinions were offered by the older adults. Many older adults have sufficient physical activity equipment in their home, but space to use it is limited.	‘I did have an exercise bike that became clothes hanger but who hasn’t used it in this way? Therefore, I sold out now I don’t have any exercise equipment here.’ (IDI-19F, British, High SES) ‘Currently my wife has put a treadmill near to dining table but I am thinking I will move the treadmill to a more conservative area so I can use it more.’ (IDI-5M, Pakistani, High SES)
	Electronic media and sedentary behaviour	Older adults mentioned that electronic devices like computers, laptops, iPads, televisions (TV) and mobile phones were often used for various sedentary activities. For some, in contrast, this provided support for their physical activity.	‘I use this smart device to do Zumba.’ (FG1-F-3, Pakistani, Middle SES)
**Policy level**	COVID-19 pandemic and health	The overarching theme was related to changes in the physical activity of older adults since the COVID-19 pandemic restrictions were implemented. Moreover, some older adults felt that they are still inactive because they are not fully recovered from the disease.	‘I was hit by the Covid-19 last year and I feel that still I am not recovered completely because I don’t feel as active as I was before.’ (IDI-14F, Pakistani, Middle SES)
	Safety concerns after COVID-19 pandemic	Even after the pandemic many older adults expressed a marked fear of leaving their home or stepping outside. They associated being at home with being secure.	‘Everything is open now, but I think it is unsafe for me to go out. I think I wouldn’t be able to go in a group setting.’ (IDI-22F, Bangladeshi, High SES)
	Diminished functional capacity after COVID-19	Moreover, due to the effects of the pandemic restrictions many older adults still find it difficult to carry out their normal day-to-day indoor activity.	‘The COVID-19 pandemic has affected me a lot because we were restricted for so long in the home. So now walking is harder for me, and it is difficult to get back again. This has certainly affected my daily routine patterns and activities.’ (IDI-10F, British, High SES)
	COVID-19 pandemic and home space	On the other hand, many older adults shared that the ability to access the garden and home space became more crucial as lockdown limitations intensified. They stated that the pandemic has forced them to be more active and do more household tasks.	‘The pandemic has made me more active; it has forced me to do more exercise because I was unable to go out to do any work out and to go to the gym. So, it’s another way around for me as compared to other people.’ (IDI-3F, African, Low SES)
	Modification of the healthcare system	Moreover, changes in the healthcare system at the policy level were also suggested by the older adults to implement physical activity initiatives. They mentioned that there is a diverse range of needs that is not being catered for.	‘What we need is differentiated activities, e.g., tai chi, dance groups, gardening, and cooking for older people who are suffering from weakness, stiffness, or pain. I think that catching problems early is better for people and cheaper for the healthcare system.’ (IDI-28F, British, Middle SES)

### Theme 1: Individual level: Attitude, perception, and motivation

#### Positive attitudes towards PA

Older adults shared that they were motivated to be active to remain mobile, fit, and healthy, avoid pain and maintain mental health and wellbeing. One older adult also verbalized to avoid being burden on family members they keep themselves busy in different activities within home. Hence these favourable PA attitudes facilitate greater PA level among older adults as reported.

*‘I keep myself mentally and physically busy; sitting down and watching television is not me. I am busy all day.’ (FG1-F-1, Pakistani, High SES)*

*‘I don’t want to be dependent on anyone or become burdened on my children, family, or anyone. This also motivates me to do physical activity.’ (FG4-F-2, Irani, Middle SES)*

#### Self-perceived health

Declining physical health act as a barrier to continuing physical activity. Some older adults reported having limited mobility due to chronic disease or age-related decline made it challenging for them to engage in physical activity.

*‘I suffered from sciatic pain and the level of sciatic pain depends on the movement or activity. I need to limit the amount of activity I do.’ (IDI-19F, British, High SES)*

*‘I feel pain in my knees during activity. Even I am unable to stand for long due to knee pain.’ (FG4-F-3, Irani, Middle SES)*

However, some older adults continued to perform their activity despite their health issues and made attempts to keep themselves moving. It was identified that some older adults overcame perception of their health as a barrier in doing physical activity.

*‘I walk throughout the day in my home. It is important for me to be active due to sciatica and arthritis; sitting in one place is not good for me.’ (IDI-22F, Bangladeshi, High SES))*

*‘I don’t call it disability; I have an ileostomy and I have a stoma bag that I have to manage during physical activity. But I don’t have issues with other parts of the body.’ (FG3-M-2, African, Low SES)*

#### Positive self-perception

Positive self-perceptions of aging were strongly linked with physical activity. Many older adults shared that positive self-perceptions assist them in being active and doing physical activity.

*‘I think age is just a number I am active and younger mentally. I don’t think myself old…I think I am active I can do everything. If you think you are old, you will never take care of yourself.’ (FG1-F-3, Pakistani, Middle SES)*

#### Lack of motivation

‘Lack of motivation’ was a theme identified in the present study. Many older adults shared that they were keen to be physically active, knowing the benefits of activity for their health, however, lack of motivation prevented them initiating physical activity within their home environment. Therefore, inadequate motivation, perceived as laziness, slowed down older adults in doing physical activity irrespective of all other facilities such as space and time as highlighted by participants:

*‘I have time but it’s laziness that is hindering me to do physical activity, I have to motivate myself to get involved in any physical activity like exercise or yoga at home.’ (IDI-20M, African, Middle SES)*

### Theme 2: Interpersonal level: Family and Friends: A motive to remain active

Family support was an important theme that was identified in this study. Living with the family had a great impact on older adults’ status of being active, functioning and performing their roles and responsibilities. Family members were perceived as a source of motivator and support system for older adults to be active within their home space. Many older adults discussed that their family members not only encouraged them to be active but also assist them in physical activity within home surrounding. Therefore, having family who supported PA promotion was one of the important determinants that helped older adults in spending less time in pursuing sedentary behaviour.

*‘My family member encourages me to do walking within the home or in the garden … my son and daughter walk by my side in the garden, so I don’t fall out during walking.’ (FG1-F-2, Pakistani, Low SES)*

*‘My daughter also encourages me to go for walk to get rid of knee pain.’ (FG2-F-2, Bangladeshi, Low SES)*

Many elderly females also shared that their family play an influencing role in assisting them in using exercise equipment or using online medium for workout, preferring to use them alongside their family member. This promotes positive physical activity environment within the family and motivates individual to remain active, as discussed by a participant:

*‘Me and my daughter go for online exercise sessions. Now we have an XXX exercise CD. We just turn on the TV and do work out for 30 minutes three times a week.’ (IDI-18F, African, High SES)*

Older adults stated that the presence of grandchildren in the home had also increased their activity level. They verbalized that spending time with grandchildren assist them to remain active and maintain their roles at the individual and familial level. This certainly promotes their physical functioning as well as have positive effect on their psychological well-being by having a sense of connection with their surroundings.

*‘In terms of health and well-being, grandchildren in the house keep me active and promote my well-being too…because they keep home noisy and active otherwise it would be very lonely. I don’t have any mental health issues because I have constant interaction with my family.’ (IDI-16F, African, High SES)*

However older adults living without family found themselves lonely and less active as shared by a participant:

*‘When my mother was there, I was active but when you are alone you become lazy.’ (IDI-20M, African, Middle SES)*

Friends’ influences apparently had a major effect on physical activity and sedentary behaviour, due to the support they give. This also provides an opportunity to have positive perception about themselves as reported by a participant.

*‘…my friend helps me to keep myself young because they say we are young and then I also consider myself young. I am still young I will do everything. I never give up.’ (FG1-F-4, Pakistani, Low SES)*

### Theme 3: Organisational Level: Health care institute

A key organisational-level theme was the role of health institutes for encouraging older adults to be physically active within their home. They shared that most healthcare organization prefer medicines instead of recommending exercises. Therefore, they suggested the need for introducing exercise prescription model in the healthcare system as it played a substantial role in optimizing their activity and minimizing their sedentary pattern within home space as seen through participant’s quote:

*‘I have experienced on my own that the exercise prescription model is fabulous.’ (IDI-30F, British, High SES)*

*‘I think more time should be invested in natural remedies or physical activities or yoga rather than just giving medications to elderly and making them dependent on them.’ (IDI-13F, Bangladeshi, Middle SES)*

Older adults discussed that the role of healthcare professional advice is very important in directing them to improve their activity level. They shared that they had been suggested by their physician or healthcare experts to do certain exercise within their home space that help them to improve their health and well-being. While some suggested the need of consulting physician to seek guidance regarding activity and workout depending on their health condition.

*‘Other than walking I also do some gentle stretches that are suggested by neurologists. My leg and pelvic muscles are weak so to make them strong I do some kinds of exercise as suggested by the doctor.’ (IDI-13F, Bangladeshi, Middle SES)*.

*‘Maybe I consult any physician so they can guide me on what outdoor activities I can join or what indoor activities I can do with devices like treadmills, exercise bikes and etc.’ (IDI-21F, Pakistani, High SES)*

### Theme 4: Community level: Significance of social groups

Being part of a social group often provided opportunities for the older adults to be physically active. This is associated with the interpersonal component of community that gave older adults the opportunity to remain connected with their peers in their home setting. However, some suggested the need for culture sensitive groups.

*‘People are alone in their homes they don’t do anything. It is like older people need to be motivated and encouraged. There should be some groups that can organize some sort of activity plans to make them more mobile within their home. There is a need of forming some local groups that help older people in doing physical activity within homes…’ (IDI-10F, British, High SES)*

*‘Culture-appropriate activities for elderly people from the west community are required.’ (IDI-21F, Pakistani, High SES)*

Lack of access to Asian community facilities was another community theme. Older adults stated that the Asian community centres would assist them to be physically active, however its unavailability lessened their opportunities to be physically active within community.

*‘Again, problem is that in Swansea we don’t have an Asian community centre but other cities like Birmingham, Manchester, and Bristol have these facilities.’ (FG5-F-1, British, High SES)*

### Theme 5: Physical environment: Microenvironment

#### Significance of the home and garden space

Older adults perceived that the home layout plays a significant role in their PA. They shared that proper space in their rooms helps them to be active and keep moving as they are able to perform different kinds of physical activities within their home environment. This PA within their home space helps to promote their positive well-being.

*‘My home is spacious, so I like to roam within home rather than sitting on a place.’ (FG2-F-2, Bangladeshi, Low SES)*

Moreover, many older adults shared that their daily household chore is a way to keep them physically active, busy and minimize their sedentary behaviour.

*‘I am more indoor person because I really like my home space as it keeps me busy doing activities within the home… I don’t like when the cleaner comes to clean the house. I like to clean the house by myself.’ (IDI-16F, African, High SES)*

Older adults admired their garden and commented that it was a major source of physical activity and rejuvenation for them.

*‘I have a garden where I do some exercises and enjoy gardening. It keeps me fit and moving. I am glad I got a garden that keeps me busy.’ (IDI-12F, British, Middle SES)*

#### Indoor and outdoor space for active and sedentary activities

Designated spaces within the home were associated with particular functions for these older adults. In addition, many older adults had dedicated indoor spaces for active, creative and sedentary activities.

*‘In the living room, I sit down and read or watch TV.’ (IDI-27M, British, Middle SES)*

Similarly, stairs within home environment serve as a mean of activity or sedentary pattern for older adults.

*‘I have stairs… I push myself to use stairs as this keep me going…’ (IDI-13F, Bangladeshi, Middle SES)*

*‘For a long time, I stay on the bottom floor because it is hard for me to get up and down the stairs in my home.’ (IDI-4M, Pakistani, High SES)*

While outdoor space is mostly used for active pastimes like cutting the grass and playing with grandchildren

*‘The basketball nets the kids play; I play with them.’ (IDI-16F, African, High SES)*

#### Changing infrastructure within the home space

On inquiring about how to make their home environment supportive of activity, many older adults shared diverse views. Some expressed possible ways to utilize their home gym effectively, while some expressed a desire to buy physical activity equipment. Interestingly, some older adults came up with the idea of designing an indoor swimming pool, dance floor, sport room, and home gym, to optimize their activity, promoting their health and well-being.

*‘…a small swimming pool within the home to be active…’ (IDI-11M, Iraqi, Middle SES)*

#### Not ready for any changes within the home environment

A few older adults highlighted their different reasons for not making any changes within their home surroundings. These included inadequate space to do so, they simply prefer not to make any changes, or, because it was developed by their parents and loved ones and they have a strong emotional attachment with the place. They mentioned that they have many memories associated with their home as they have spent their entire life there with their loved ones.

*‘… my mom has kept home in a way for years and it has just been placed in that way… I love every part of my home …as good memories are attached to it. It is the place where me, my father, uncle, and my mother spend the good days of our life.’ (IDI-21M, Pakistani, High SES)*.

On the other hand, there were also restrictions imposed by some older adults’ family members, some of which impacted on the participant’s ability to be mobile. Their reluctance to make modifications to the home included not liking change but also for aesthetic reasons.

*‘I need a mobility toilet because I got arthritis. I need to put a mobility aid, but my family has not allowed me because they do not want to spoil walls.’ (FG5-M-3, British, High SES)*.

#### Physical activity equipment

In terms of availability of physical activity equipment within the home mixed opinions were offered by the older adults.

*‘I did have an exercise bike that became clothes hanger but who hasn’t used it in this way? therefore, I sold it out now I don’t have any exercise equipment here.’ (IDI-19F, British, High SES)*.

On the other hand, many older adults have sufficient physical activity equipment in their home, but space to use it is limited.

*‘Currently my wife has put a treadmill near to dining table but I am thinking I will move the treadmill to a more conservative area so I can use it more.’ (IDI-5M, Pakistani, High SES)*

#### Electronic media and sedentary pattern

Older adults in this study mentioned that electronic devices like computers, laptops, iPads, televisions (TV) and mobile phones were often used for various sedentary activities. Many older adults stated that social media was also one of the reasons for using mobile phones although they were well-informed about the potential unhealthy effects of using these devices. Moreover, they have not set any rules for themselves in relation to media use, resulting in increased SB.

*‘No, I don’t have made any rules for myself around media. It is like I have never noticed time. I never notice at what time I start using iPhone and at what time I stopped.’ (IDI-7F, Pakistani, Middle SES)*

For some, in contrast, this provided support for their physical activity.

*‘I use this smart device to do Zumba.’ (FG1-F-3, Pakistani, Middle SES)*

### Theme 6: Policy level

#### COVID-19 pandemic and health

The overarching theme was related to changes in the physical activity of older adults since the imposition of COVID-19 pandemic restrictions were implemented. Moreover, some older adults felt that they are still inactive because they are not fully recovered from the disease.

*‘I was hit by the covid-19 last year and I feel that still I am not recovered completely because I don’t feel as active as I was before.’ (IDI-14F, Pakistani, Middle SES)*

#### Safety concerns after COVID-19 pandemic

Even after the pandemic many older adults expressed a marked fear of leaving their home or stepping outside. They associated being at home with being secure.

*‘Everything is open now, but I think it is unsafe for me to go out. I think I wouldn’t be able to go in a group setting.’ (IDI-22F, Bangladeshi, High SES)*

#### Diminished functional capacity after COVID-19

Moreover, due to the effects of the pandemic restrictions many older adults still find it difficult to carry out their normal day-to-day indoor activity and it is persistence even after the pandemic.

*‘The COVID-19 pandemic has affected me a lot because we were restricted for so long in the home. So now walking is harder for me, and it is difficult to get back again. This has certainly affected my daily routine patterns and activities.’ (IDI-10F, British, High SES)*

#### COVID-19 pandemic and home environment

On the other hand, many older adults shared that the ability to access the garden and home space became more crucial as lockdown limitations intensified. They stated that the pandemic has forced them to be more active and do more household tasks.

*‘The pandemic has made me more active; it has forced me to do more exercise because I was unable to go out to do any work out and to go to the gym. So, it’s another way around for me as compared to other people.’ (IDI-3F, African, Low SES)*

Many older adults performed different physical activities such as exercise, dancing and walking within home setting due to proper space in the home and presence of garden.

*‘It is strange I retired just as COVID-19 started. I do a lot of gardening and build a tango floor. I think I am fortunate that we don’t live in a small house or flat without a garden which is awful. For us, it was fine because we were busy in our house and garden.’ (IDI-28F, British, Middle SES)*

#### Modification of the healthcare system

Moreover, changes in the healthcare system at the policy level were also suggested by the older adults to implement physical activity initiatives. They mentioned that there is a diverse range of needs that is not being catered.

*‘What we need is differentiated activities for, e.g., tai chi, dance groups, gardening, and cooking for older people who are suffering from weakness, stiffness, or pain. I think that catching problems early is better for people and cheaper for the healthcare system.’ (IDI-28F, British, Middle SES)*

Moreover, many older adults shared the need of substantial improvement in the healthcare system. In addition, they discussed that lack of investment in the healthcare sector results in minimizing initiative for older adults’ physical activity. Therefore, older adults suggested the need of funding assistance for physical activity projects. This will also call for more liaison between the fitness industry and health care professionals.

*‘Some kind of financial help or grant towards the cost of taking part in an exercise class where appropriate. Possibly more communication or coordination between the fitness industry and health professionals.’ (IDI-29F, British, Middle SES)*

Further they also shared that healthcare system should encourage people to be active from a young age which would also be one of the beneficial strategies for making people active in later stage of life.

*‘Strong steps for prevention of disease rather than treatment of disease. It’s like the British healthcare system should work actively with all age groups. If you encourage people from an early age to be active and enjoy physical activity and join social groups probably when they reach to my age, they will make a healthy community of older people.’ (IDI-27M, British, Middle SES)*

## Discussion

This study explored older adults’ perception of their home environment and its impact on their PA and SB using the SEM as a theoretical framework. The findings indicate that several factors at different levels of the SEM had an impact on older adults’ PA and SB. These include: 1) Individual level: Attitude, perception and motivation 2) Interpersonal level: Family and Friends: a motive to remain active 3) Organisational level: healthcare institutes, 4) Community level factors: Significance of social groups, 5) Physical Environment: Microenvironment and 6) Policy level factors (lockdown restrictions and healthcare system) impacting older adults’ PA and SB at home.

Within the individual level of the SEM model factors such as being lazy, poor health or belief that being physically active was a crucial component of independence all contributed to older adults’ PA and SB in their homes. Existing activity habits and the development of activity routines within the home environment have been noted to be significant drivers of participating in PA in older adults [66, 67]. Moreover, awareness of the mental and physical health benefits of being physically active can motivate older adults to remain active and moving, whereas poor health can act as an obstacle. This supports previous findings on how to encourage older adults to be physically active [67–69]. In addition, positive views on ageing also foster PA among older adults despite their poor health. This finding parallels those of earlier studies showing that older adults with positive self-perceptions of ageing engaged in more PA such as walking and playing sports [70, 71]. In addition, it was also noted that older adults with poorer health who had positive perceptions of ageing walked the same amount as with good health [70]. Hence underscore the importance of positive perceptions and extend understanding of how they also influence home-based PA and SB.

The current findings illustrate the dynamic nature of the home and the interactive effect of different factors at all levels of the SEM on older adults’ PA and SB, which is consistent with prior research [72–74]. The physical environment of the home can act as both a hindrance to, and facilitator of, PA and SB, however, as shown in earlier research [77, 79], our findings indicate that the family within the home modifies the environment dynamics. This was clearly evident in our findings as families were sometimes responsible for making or restricting modifications that helped or hindered these older adults’ PA and SB at home. In addition, different home social environments, such as living with family or alone can act as either an enabler and barrier to PA and SB. This is in line with a systematic review that suggested that family is the most crucial factor to take into account when examining sedentary pattern and PA behaviour and attitudes [75]. Moreover, prior research found that grandchildren engaging older adults in PA has been strongly linked with higher activity levels [76, 77]. Similarly, in the present study, spending quality time with grandchildren appeared to enhance older adults’ PA and minimize their SB through structured and unstructured play, which has numerous advantages for older adults’ physical, mental, and social wellness [77].

The social component of participating in activities is therefore a key motivator for maintaining PA and minimizing SB within the home environment and engaging in group activities helps to build and maintain relationships with others. Previous research studies [78–80] have highlighted that having opportunities for making social connections with friends encourages older adults’ participation in physical activities. However, in our study, many Muslim female older adults emphasized the need for culture sensitive social groups. Similarly, in previous research it was identified that Muslim women are discouraged from participating in mixed gender activities because of personal or familial gender-based expectations and modesty [81]. Therefore, our findings reinforce earlier suggestions that social facilities that are consistent with the cultural beliefs and practises of these groups must be provided to ensure the involvement of diverse communities [81, 82]. Doing so can offer an incentive for these older adults to be active in their homes as a platform to engaging in community-based physical activities.

At the organizational level healthcare institutes play a significant role in encouraging older adults to be physically active and avoid SB within their home. Evidence shows that motivation is a crucial driving force for older adults to participate in physical activity and increase their participation in PA programs [83]. Current findings showed many older adults feel motivated and completed exercises when they were prescribed by healthcare professionals and accounted for their physical limitations and abilities. Literature also suggests that the most effective way to spread strategies is through health care professionals, who can offer guidance and motivation on what to do based on the health status of each older person [84]. Therefore, healthcare organizations should motivate healthcare professions to adopt this culture of being advocates for PA and reducing SB as this could play a significant role in improving the attitudes of older people toward active living in their homes and daily routines.

The indoor or microenvironment also plays a key role in older adults’ PA and SB. It was found that large spaces within the home environment, such as spacious rooms and a garden, enabled older adults to perform different kinds of physical activities. This study finding is consistent with the literature emphasizing that homes with broader corridors along with multiple rooms or clearly defined spaces facilitated activity among older adults [59]. In addition, it has been reported that outdoor space (i.e., a garden) was associated with the highest levels of PA and self-reported physical health among older adults [85]. This enabled them to expend varied amounts of energy, based on the compendium of PA for metabolic equivalent (MET) intensity levels [86]. Hence making the home conducive to promote transition (sit-to-stand), as highlighted in the 24-Hour Movement guidelines that emphasize the need to reduce SB to ≤ 8 hours [54]. Moreover, the ability of individuals to directly manage key aspects of the physical environment within the home appears to set the home apart from other settings that aim to encourage individuals to be physically active. This finding illustrates the importance of the person-environment interaction, as espoused by a SEM approach, in influencing behaviour, in this case, PA and SB [87].

The possession of PA equipment, along with its accessibility, was another significant factor within the home. The availability of environmental and PA resources can enhance the likelihood of individuals performing PA and act as an initial step in meeting the recommended level of PA [88, 89]. Accessibility is associated with the feasibility of use and cueing of behaviour, as activity or exercise equipment that is instantly available with convenient access requires less effort to use compared with that which is less conveniently located [90], making this a crucial factor for PA. However, family members often moved equipment to an inaccessible place within the home environment, thus decreasing older adults’ PA at home environment. This highlighted that various factors at different levels of the SEM interact within the home physical environment to influence older adults’ SB and PA behaviour. Further, there is a strong connection between physical and social environments [39, 91], which may act synergistically causing an impact on the individual’s behaviour [39]. Therefore, it is arbitrary or illogical to separate physical and social aspects of the environment [92].

Electronic media use within the home environment plays a significant role in older adults’ PA and SB. Many older adults in this study spend a substantial amount of time on social media though they realized that this could have implications for their health and well-being. Being autonomous and without any restrictions within the home, many older adults do not set any rules around media use, resulting in long hours spent on these devices and long periods of inactivity. In contrast, a few older adults utilized technology to perform PA within the home. This shows that autonomy allows the individual freedom to carry out behaviours of their own choosing as highlighted in self-determination theory [93].

Many older adults suggested that restructuring or modifying the physical environment within the home assists them in performing PA within their home as well as enhances their autonomy, freedom, integrity, and independence within their home [94, 95]. Interestingly, many older adults suggested quite substantive modifications such as installing an indoor dance floor, swimming pool, sports and gym room, or purchasing PA equipment, such as a treadmill, to make their home environment conducive for PA. However, this significant modification would certainly require significant resources, time and finance. Therefore, the study findings supported and recommended the notion of ‘think small for large effects’ within the home space that helped older adults to map out small-scale activities to support their daily life-style activities like keeping PA devices in a convenient place, avoiding screens in bedrooms, or using the phone in only one room as compared to large-scale less realistic infrastructure changes. This also presents that SEM places a strong emphasis on the role of the physical environment and its interaction with diverse factors.

In contrast, many older adults were reluctant to make any changes to support increases in PA because they simply prefer to maintain the status quo, or, because their home environment was fashioned by their parents and loved ones and they were emotionally attached to it. Previous literature has also highlighted that the ageing process and ingrained memories increase an older adult’s sense of attachment, and the desire to remain in and age in one’s current home [96, 97]. Moreover, as suggested in past studies, family members within the home play an influential role in changing the environment [98]. This was poignantly made clear in our study as restrictions imposed by family members presented a major hindrance to modify home surroundings for some participants. If the home is not well adapted, this limits older adults’ mobility inside their home, particularly as people get older, they feel more vulnerable and endangered, therefore they seek greater safety and harmony [99] which means they are more likely to confines of their own home. Hence, the interaction of individual and social factors along with the environment of the individual play an important role in enhancing PA and minimizing SB, as described in the SEM of health behaviour [100–102].

Our findings suggest that there is still much to do at a policy level to motivate older adults to adopt PA within their lifestyle. The views of the older adults suggest that there was an increase in SB and a decrease in PA due to the imposition of the lockdown restrictions, which has previously been reported [103, 104]. This decline in PA might be due to isolation related to the pandemic [104, 105], as many older adults in our study reported being lonely, spending more time in sedentary activities like social media use, and watching TV and less time participating in PA. The restrictions meant that the social components that motivate many older adults to take part in activity were no longer available and still confine their activity even after the restrictions have been lifted. This highlights the need for policies to minimize older adults’ SB and enhance their PA within their home environment. Hence, the COVID-19 regulations created a restrictive environment that limited opportunities for older individuals to be active, hindering autonomy, competence, and relatedness, highlighted as key determinants of motivation by self-determination theory [93]. In the current study, older adults experienced a lack of autonomy in their choice to be physically active, had few opportunities to develop or experience competence in being active, and were unable to experience relatedness when being active, that for many older adults is a primary motivator. However, a few participants had increased their usage of their home and garden space for activity as a result of the lack of opportunity to engage in activity elsewhere and their experiences might offer valuable insight to inform such future policy.

As our findings illustrate, the previously described facilitators and inhibitors of PA and SB within the home are multifaceted and cannot be comprehended or addressed if different layers of the model are examined separately. Using these findings to develop interventions to address older adults’ home-based PA and SB suggests that accounting for the interactions, reciprocal associations, and dynamic nature of relationships between the factors, is most likely to produce effective outcomes for changing behaviour. For instance, one such interaction involves an older adult’s motivation and attitudes towards PA and SB at the individual level with a number of interpersonal factors, such as support from family and friends. It is also important to take into account interactions within a level, for example, the interaction between the individual factors of motivation and self-perceived health. It is well established that deteriorating health can demotivate older adults to be active [106].

When interpreting the present study findings, study strengths and limitations should be considered. This study has contributed to the evidence base on older adults’ home-based PA and SB by comprehensively using all levels of the SEM to interpret the facilitators of, and barriers to, reducing SB and increasing PA. Moreover, both individual interviews and focus groups with multi-diverse older adults allowed for wide-ranging discussions and enable in-depth exploration of their PA and SB and attitudes in regard to their home environment. However, a number of limitations should be taken into account in the current research. First, the size of typical UK home is decreasing [107] and this may have influenced older adults’ perceptions of their home space and garden. Second, older adults were recruited from low, middle and high socioeconomic (SES) backgrounds in an effort to ensure equal representation. However, high SES older adults overrepresented in our sample, irrespective of this over-representation the current study finding may be generalisable to areas which have similar home physical environment features.

## Conclusion

The dynamic association between the diverse factors at different layer of SEM and home environment highlighted both the facilitators and barriers for older adults PA and SB that can be taken into consideration for optimum engagement. This suggests that the home environment cannot be addressed in isolation as it is complex and is influenced across different levels of the SEM. Therefore, interaction between these layers has given us an integrated perception of the older adults PA and SB within their home space.

To better understand how to promote PA and minimize SB in older adults’ home environments, a dynamic systems approach must take into account by bringing changes in these interactive determinants to intervene effectively. This will motivate older adults to develop intentions that reflect positive perceptions of control over their home environment which is significant for promoting PA.

## Supporting information

S1 File Interview guide. (DOCX)
