# Supplementary material for: Older adults’ perspectives on physical activity and sedentary behaviour within their home using socio-ecological model
Source: PLoS One. 2023 Nov 20;18(11):e0294715. doi: 10.1371/journal.pone.0294715 (PMC10659182; doi:10.1371/journal.pone.0294715)
Supplement: S1 File — (DOCX) [file pone.0294715.s001.docx]

**Interview Guide**

**Applied Sports Technology Exercise and Medicine Research Centre (A-STEM)**

College of Engineering

**Project Title:**

Older adults’ perspectives on physical activity and sedentary behaviour within their home using a socio-ecological model.

**Study Information**

| **information collected by:** | Naureen |
| --- | --- |
| **Today’s Date:** |  |

**Contact Information**

| **ID** |  | | |
| --- | --- | --- | --- |
| **address with postal code** |  | | |
| **neighborhood/community** |  | | |
| **telephone numbers** | **home:** | **cell** | **neighbor/relative** |

**Demographic Information**

| 1.Gender |  |
| --- | --- |
| 2. Age |  |
| 3.Your highest education level | 1. No formal education 2. Less than primary school 3. Primary school completed 4. Secondary/High school completed 5. College/University completed 6. Post graduate degree |
| 4. Your occupation | 1. Government employee 2. Non-government employee 3. Self-employed 4. Non-paid 5. Retired 6. Unemployed (able to work) 7. Unemployed (unable to work) 8. Other, specify:  ____________ 9. ____________________________ |
| 5. What is your nationality? | 1. Pakistani 2. Indian 3. Chinese 4. Bangladeshi 5. African 6. White 7. Other____ |
| 6. What is your current marital status? | 1. Married 2. Living as couple (living together, but not legally married) 3. Divorced or separated 4. Single, never married 5. Widowed 6. Other (Specify): _______________________________________ |
| 7. Current House Status | 1. Owned  2. Rented  3. Other_________ |

Thank you for agreeing to take part in this study. We are interested in finding out more about what you do to keep physical activity. In the interview I will ask you about physical activity; by this I mean any movement of your body which requires you to use energy. This includes exercise you might do, for example, swimming or walking for fitness, but it also includes other types of activity such as gardening, housework, dancing, DIY, walking to the shops etc. If you are not sure what any of the questions mean, please let me know.

1.How are you right now? How are your currently feeling?

2. Some people describe themselves are being 'outdoorsy' or “indoorsy’ people. How would you describe yourself? Prompt: Would you say you are more indoorsy or outdoorsy? Do you like being active like walking in your spare time? Or doing things like watching TV, being on the computer or reading?

3.Why do you say that? What makes you choose to do something active like walking in the garden vs watching TV?

4.Can you please tell me about your thoughts regarding physical activity like benefits on health?

5. What kind of activities do you perform at home?

6. What opportunities do you have to be physically active within home evironment (e.g place to walk within home/ outside home/ in the garden, indoor activities table tennis, exercises, Yoga, Tai Chai, walking, gardening and cycling)

7. What motivates or promotes you to do physical activity within home/ garden (exercise with someone, Knowledge, Fear and Negative Experiences, Disease Management, Social Isolation, Advice from healthcare professional Social Activity, Physical and mental health / wellbeing, Environment, Time,weather etc)

8. Do you have proper support from your family to do physical activity at home?

9. Any other factors that might influence your PA level within home?

10. What prevents you from doing physical activity in your home setting? (space, disease, time, health, weather and isolation).

11. Do you experienced any pain whilst being physically active?

12. Do you have any fear that physical activity might worsen your health rather than improve health conditions (e.g. arthritis, respiratory disease, or symptoms like angina for example)

13. How do you feel about using technology like mobile phone apps, exercise videos/DVDs or exercise games/ steps count watch/virtual personal trainer/virtual workout groups to help you keep physically active?

**Thinking about your home:**

1. What are your favourite features of the house that you most like and why? Do you have garden what do you like in the garden?
2. What do you most dislike in the house and garden and why?
3. Why did you choose this home for your family/yourself to live in? What things did you consider?
4. Why is your home organized in the way that it is at present?
5. Have you changed anything about your home over the past 5 years (e.g., technical equipment or device and the reason of these changes in home?
6. If you could change anything about your home, what would it be? Why would you do this?
7. What if you could redesign your house to do things like rearrange the rooms or make areas bigger or smaller? What would you do?
8. Do you have access to mobile technology like laptops, smart phone, handheld electronic game etc? Where do you usually use these at home? Where do you usually access the internet at home?
9. Can you tell me the time spent watching TV, playing video and computer games, and using the internet for leisure, on a typical week and weekend day?

1. What if you had to make changes to the house and garden to make it more active? What would you do?
2. What if you had to make changes to the house & garden to get away from screens and spend less time on the couch? What would you do?
3. What do you think that currently your home physical environment (example space within home) promotes your health and well-being? (Prompt why? What are the factors?)
4. Are there any parks/gyms nearby home and how frequently do you go there?
5. Are there any community physical activity services for older people nearby your home?
6. Do you have any physical activity equipment (treadmill, exercise bike, weights/resistance band) within your home?
7. What do you think that pandemic has restricted your physical activity level within your home surroundings? (Prompt: Is there any change in daily routine)

**Recommendations:**

1.What do you think could help people maintain a healthy lifestyle as they get older?

2.In order to help older people to be physically active, what services/initiatives do you think should be added/improved in the community?

3.What are your suggestions and recommendations for older people in terms of improving their home space to be more physically active?

4. Do you like to see any changes in the British health care system to improve the activity in old age life that will enhance their quality of life?
